# Ingestion of fishing gear and *Anisakis* sp. infection in a beached Indo-Pacific finless porpoise (*Neophocaena phocaenoides*) in the Jeju Island, Republic of Korea: findings from post-mortem computed tomography and necropsy

**DOI:** 10.1186/s12917-024-04090-z

**Published:** 2024-05-27

**Authors:** Sung Bin Lee, Adams Hei Long Yuen, Sunmin Kim, Won Joon Jung, Do-Gyun Kim, Sang Wha Kim, Young Min Lee, Dasol Park, Han Seok Cho, Cherry Tsz Ching Poon, Sang Guen Kim, Sib Sankar Giri, Su Jin Jo, Jae Hong Park, Mae Hyun Hwang, Eun Jae Park, Jong-pil Seo, Seongjun Choe, Gun Wook Baeck, Byung Yeop Kim, Se Chang Park

**Affiliations:** 1 Laboratory of Aquatic Biomedicine, College of Veterinary Medicine, Research Institute for Veterinary Science, Seoul National University, Seoul, Republic of Korea; 2 Radiotherapy and Oncology Centre, Gleneagles Hospital Hong Kong, Wong Chuk Hang, Hong Kong SAR, China; 3 Department of Parasitology, Parasite Research Center and International Parasite Resource Bank, School of Medicine, Chungbuk National University, Cheongju, Republic of Korea; 4 Department of Marine Biology & Aquaculture, Institute of Marine Industry, Marine Bio-Education & Research Center, College of Marine Science, Gyeongsang National University, Tongyeong, Republic of Korea; 5 College of Veterinary Medicine, Institute of Veterinary Science, Kangwon National University, Chuncheon, Gangwon, Republic of Korea; 6 Cetacean Research Institute, National Institute of Fisheries Science, Ulsan, Republic of Korea; 7 College of Veterinary Medicine, Seoul National University, Seoul, Republic of Korea; 8 Department of Surgery, Queen Mary Hospital, Pokfulam, Hong Kong SAR China; 9 Department of Biological Sciences, Kyonggi University, Suwon, Republic of Korea; 10 College of Veterinary Medicine and Veterinary Medical Research Institute, Jeju National University, Jeju, Republic of Korea; 11 Department of Marine Industry and Maritime Police, College of Ocean Science, Jeju National University, Jeju, Republic of Korea

**Keywords:** Marine litter, Foreign body ingestion, Fishing hooks, Ulceration, Diagnostic imaging techniques, Parasites, Cetacean

## Abstract

**Background:**

Human fishing activities have significantly affect environmental concern for marine ecosystems, conservation of marine mammals, and human health. Coastal cetaceans are highly vulnerable to ingestion of fishing gear, bycatching, or entanglement, all of which can be fatal for these animals. In particular, certain coastal dolphins and porpoises are heavily impacted by fishing gear such as angling gear or stownet, as their food often overlap with the target fish species of human fisheries.

**Case presentation:**

This study presents a case of an Indo-Pacific finless porpoise (*Neophocaena phocaenoides*) beached on the coast of Jeju Island, Republic of Korea, with ingestion of fishing gear and severe *Anisakis* infection. Although this species inhabits waters ranging from the Persian Gulf to Taiwan, several stranded carcasses have been reported on Jeju Island in recent years. Post-mortem computed tomography revealed a bundle of four fishing hooks in the forestomach, along with nylon lines and steel lines with connectors, which were assumed to be angling gear for Jeju hairtail (*Trichiurus lepturus*). Further necroscopic investigation revealed that the forestomach contained a large number of *Anisakis* spp. (Nematoda: Anisakidae). Histological examination revealed a thickened forestomach wall with pinpoint and volcanic ulcerations, a thickened layer of stratified squamous epithelium, and infiltrated stroma in the squamous epithelium.

**conclusions:**

This study emphasizes the urgent need to address the impact of fishing activities on marine mammals, marine litter pollution, and the bycatch problem in Korean seawater. In addition, the occurrence of *N. phocaenoides* in seawater around Jeju Island should be raised in future geographical ecology or veterinary pathology studies and when its distribution is updated.

## Background

Marine animals’ depredation can lead to the ingestion of fishing gear and entanglement, resulting in adverse effects on marine ecosystems and the conservation of marine animals making it a significant environmental concern [1–6]. Abandoned, lost, or discarded fishing gear (ALDFG) is also considered one of the most harmful threats to ocean ecosystems because of its impact on and behavior in marine habitats [7]. Globally, a significant proportion of all fishing gear is abandoned or lost annually, including 29% of fishing lines, 8.6% of traps, and 5.7% of fishing nets [8]. It is estimated that 38,535 tons of gill nets and 11,436 tons of traps are discarded or lost annually from the coastal seawaters of South Korea alone [9].

ALDFG poses a significant threat to the top predators in marine ecosystems, such as marine mammals, reptiles, elasmobranchs, and birds, in several ways [10–12]. Coastal cetaceans, including several dolphin and porpoise species, are particularly vulnerable to ingestion of fishing gear [13] and being bycaught [14] because of their proximity to the coastline and their prey species targeted by human fisheries. In general, entanglement or marine debris can be more fatal in deep-diving pelagic cetaceans [15, 16]. Because of the potential for these threats to reduce and disrupt populations of keystone species, they can have significant ramifications for entire marine ecosystems [17].

This study presents the case of an Indo-Pacific finless porpoise (*Neophocaena phocaenoides*) beached on the coast of Jeju Island, Republic of Korea, with ingestion of fishing gear and severe *Anisakis* sp. infection. Given the indications of suffocation, the specimen was also presumed to have been caught, and was considered to have perished because of two distinct human activities: abandoned marine litter and bycatch. Although this species is known to inhabit waters from the Persian Gulf to Taiwan, excluding Korean waters [18], several stranded carcasses have been reported on Jeju Island and the Korean peninsula [19] in recent years. Further studies are necessary to explain the reasons for this distributional anomaly.

## Case presentation

**Fig. 1:**
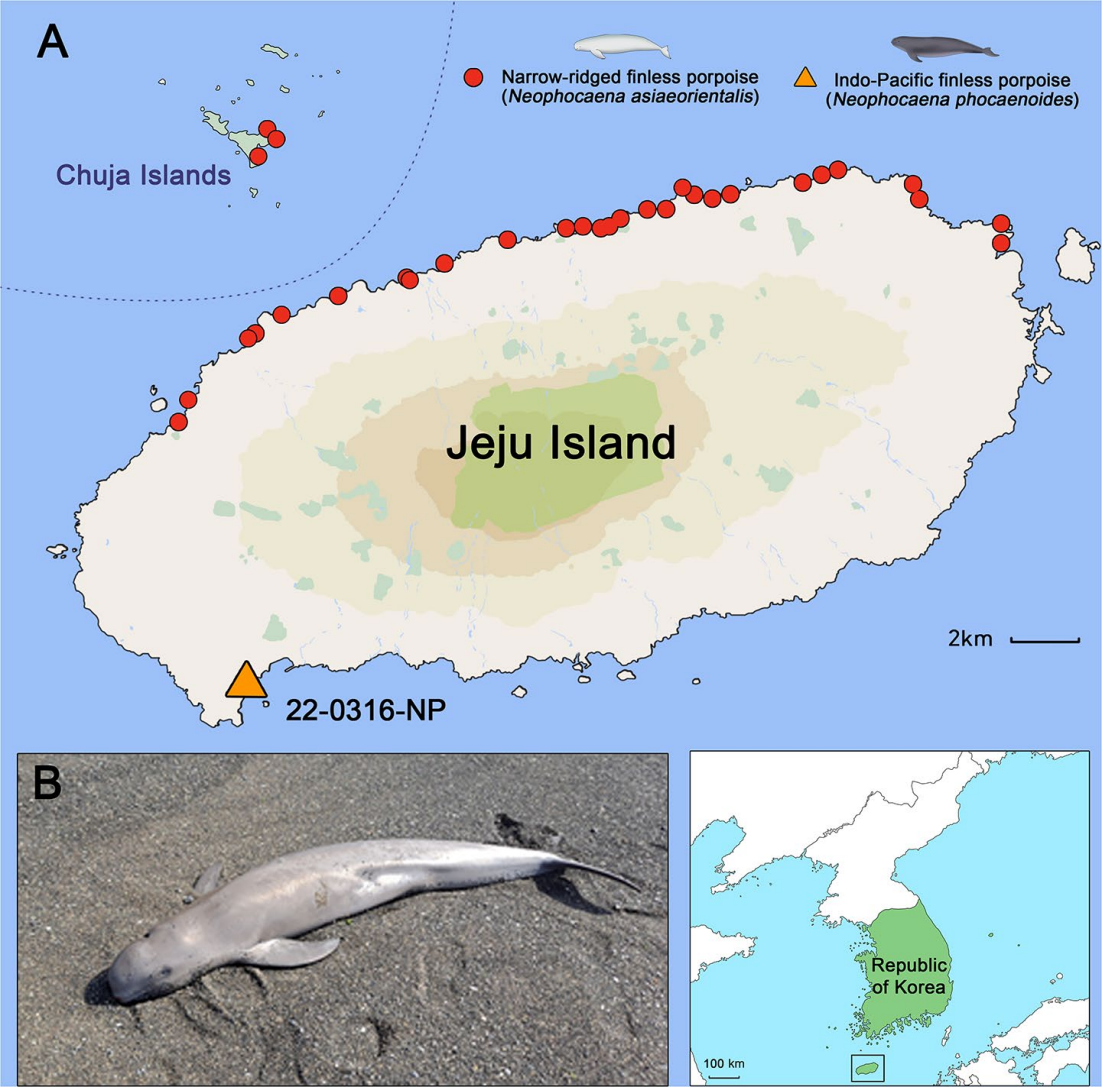
The beached location of specimen 22-0316-NP; Indo-Pacific finless porpoise (*Neophocaena phocaenoides*) and narrow-ridged finless porpoises (*N. asiaeorientalis*) during 2022-2023. (**A**) The carcass 22-0316-NP was discovered on Sagye Beach, Sagye-ri, Andeok-myeon, Seogwipo-si, Jeju-do, Republic of Korea (33°13’25.0”N 126°17’54.2”E). The dominant species in Korea, the narrow-ridged finless porpoises (red circles), are mostly found beached in the northern area of Jeju Island and around Chuja Island. However, the Indo-Pacific finless porpoise; 22-0316-NP (yellow triangle), was unusually found beached on the southeast coast of Jeju Island. (**B**) The carcass was fresh with Smithsonian condition code 2

On March 16, 2022, an adult female Indo-Pacific finless porpoise (length from snout to tail 171.0 cm) was stranded on the southern coast of Jeju Island in the Republic of Korea (33°13’25.0”N 126°17’54.2”E; Fig. 1A). The carcass was fresh (Smithsonian condition code 2) [20], and no scavengers were found upon retrieval (Fig. 1B). To minimize post-mortem changes, the carcass was immediately transported to the deep freezer at Jeju National University upon retrieval and frozen at − 22 °C until further examination for 4 months.﻿

Post-mortem computed tomography (PMCT) was performed at the Jeju National University Equine Hospital using an Aquilion Lightning 16-row, 32-slice helical CT system (Aquilion Lightning, Canon Medical Systems, Otawara, Japan) on July 17, 2022. Acquisition parameters were set at 120 kVp, 200 mAs, 1 mm slice thickness, with a scanned field of view (sFOV) of 320 mm. The obtained PMCT images were evaluated using the open-source Digital Imaging and Communications in Medicine (DICOM) viewing software, Horos version 3.3.6 (RRID: SCR_017340), performing multiplanar reconstruction and three-dimensional (3D) volume rendering of the DICOM images. The PMCT procedures and image interpretation were conducted by a board-certified radiographer (AHLY) with over 7 years of experience in post-mortem cetacean imaging.

**Fig. 2:**
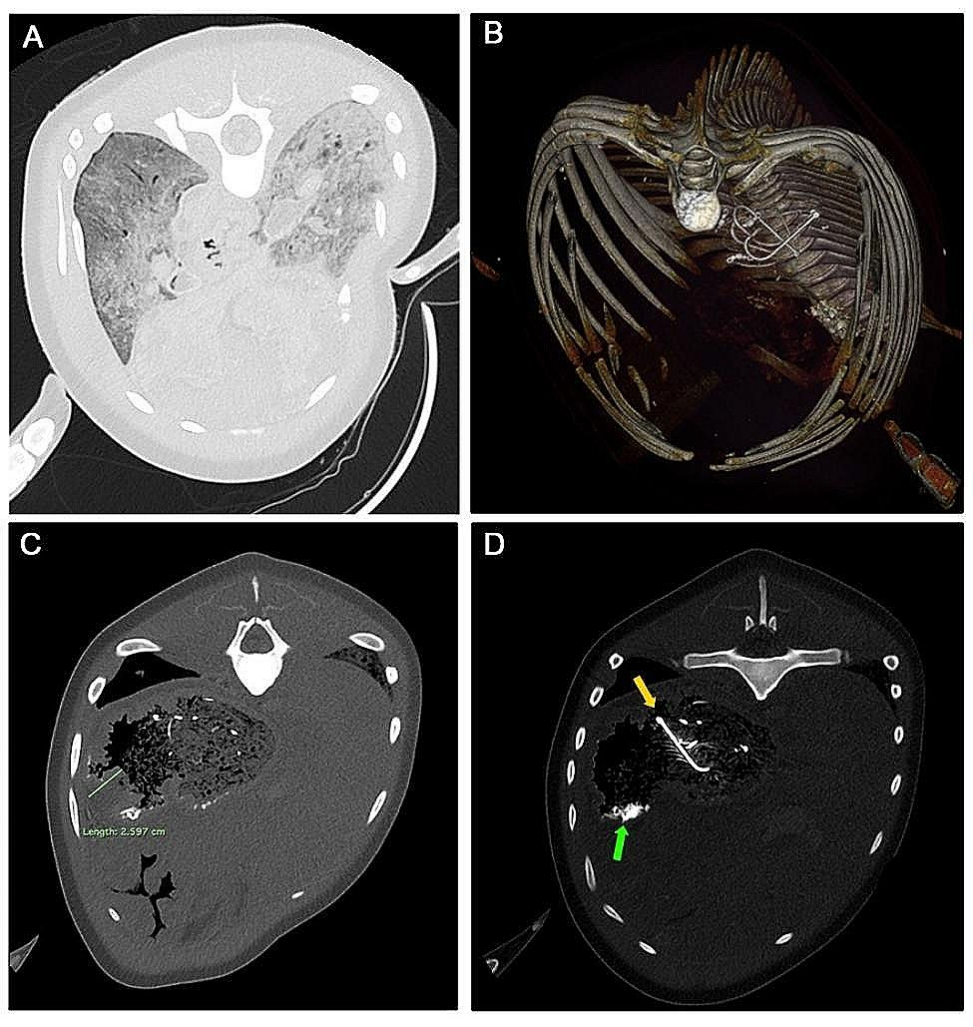
Postmortem computed tomography (PMCT) images of the specimen. (**A**) A widespread ground-glass opacity pattern is observed in both lungs. Consolidation is observed on the dorsal aspect of the left lung. (**B**) Foreign bodies presumed to be fishing gear, including four hooks, three ring connectors, and wires. (**C**) Thickened forestomach wall with maximum width of 2.6 cm. (**D**) A hyperattenuated foreign body presumed to be a mass of the parasite (green arrow) with a fishing hook (yellow arrow) in the forestomach

PMCT images (WW/WL: -500/1400) revealed widespread ground glass opacification (GGO) in both lungs forming a mosaic pattern (Fig. 2A), with a predominant presence in the left lung, indicating signs of pneumonitis. A three-dimensional (3D) reconstructed image illustrated foreign objects, including four fish hooks, three ring connectors, fishing lines, and fishing wires, in the forestomach (Fig. 2B). Thickening of the forestomach wall (typically less than 1.7 cm in the healthy adults), with a maximum width of 2.6 cm, was observed, indicating possible gastritis during the antemortem period (Fig. 2C). Parasite agglomeration was also observed in the forestomach, along with hyperattenuating foreign objects (Fig. 2D).

Necropsy of the carcass was conducted on July 18, 2022, at the Jeju office of the Korean Fisheries Resources Agency (FIRA; 23, Ongpo 7-gil, Hallim-eup, Jeju, Korea). The skin and muscle samples were collected for genetic analysis. Various organs and lesions, including those discovered in the stomach, lungs, kidneys, liver, lymph nodes, and external genitalia, were appropriately sized and collected for histological and pathological examination. All parasites were isolated from several organs, including the lungs, liver, gastrointestinal tract, and mammary glands. Samples were stored in 70% ethanol for molecular analysis and/or in 10% neutral-buffered formalin for histopathological examination.

**Fig. 3:**
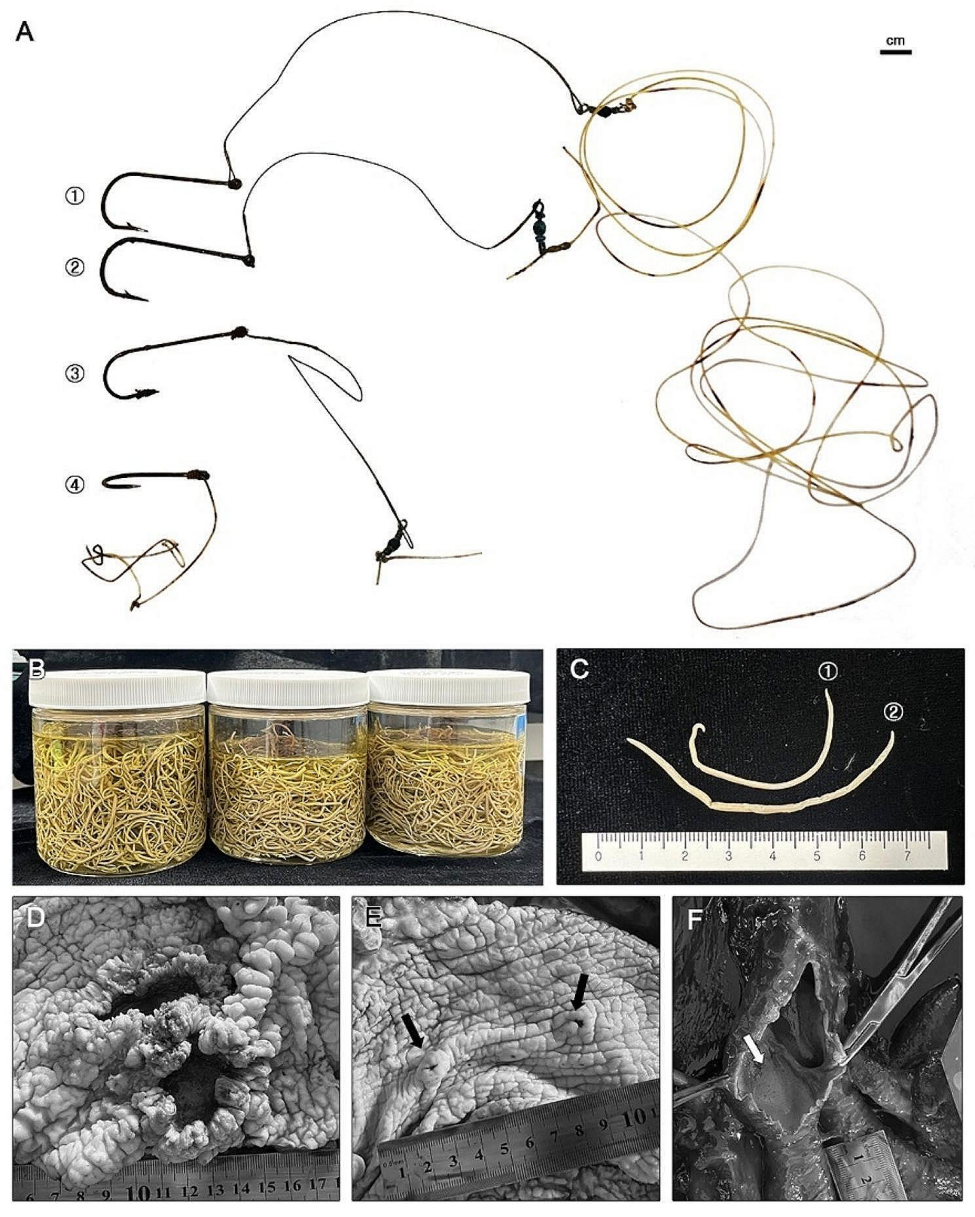
Necropsy examination of the specimen. (**A**) Foreign bodies, consisting of a bundle of four fishing hooks in the forestomach, along with nylon lines and steel lines with connectors: ① 1.8 × 4.0 cm hook with 16.0 cm steel line and 182.2 cm nylon line; ② 2.8 × 4.5 cm hook with 15.0 cm steel line; ③ 1.6 × 4.0 cm hook with 15.3 cm steel line; ④ 1.4 × 2.3 cm hook with 23.3 cm nylon line. (**B**) Parasitic *Anisakis* sp. specimens recovered from the host’s oral cavity, esophagus, and forestomach (from L to R); specimen code 22-0316-NP. (**C**) Sexual distinction in identifying *Anisakis* sp.: ① Male specimen; ② Female specimen. (**D**) Severe volcanic ulcerations in the forestomach. Due to *Anisakis* sp. superinfection, the epithelium had a punched-out appearance with thickened mucosa and chronic ulcerations by the attached nematodes on the center. The diameter of the lesion was 7.5 cm and 5.0 cm, respectively. (**E**) Pinpoint-sized aphthous ulcerations in the forestomach with thickened mucosa. In total, nine lesions were identified. (**F**) Foamy fluid that filled the respiratory tract

The carcass was extremely emaciated, with a body condition score (BCS) of 2 [21]. During necropsy, the porpoise was observed to have ingested four fishing hooks, along with nylon and steel lines with connectors (Fig. 3A). The longest line measured 182.2 cm, and the largest hook measured 2.8 × 4.5 cm.

A substantial number of nematodes, including those in the forestomach, esophagus, and oral cavity, were found in the digestive tract, causing severe ulcerative granulomatous gastritis (Fig. 3B and C). The nematode responsible for the infection was identified as *Anisakis* spp. (Nematoda: Rhabditida: Anasakidae) by morphological examination [22]. In addition, two volcanic ulcerations (Fig. 3D) and nine pinpoint-sized aphthous ulcerations (Fig. 3E) were observed on the gastric wall of the forestomach. Remarkably, the intestines were devoid of any content, suggesting an influence from gastric foreign material and appetite loss. The respiratory tract, encompassing the trachea, bronchi, and lungs, contained a foamy fluid (Fig. 3F).

Lesions that had been fixed in formalin were appropriately sized and processed by the Korean Vet Lab (Seongnam, Republic of Korea). These samples were embedded in paraffin and sectioned into 5 μm slices. Hematoxylin and eosin (H&E) staining was performed on tissue sections to detect histological changes. All tissue slides were analyzed by veterinary pathologists at Antech Diagnostics (Fountain Valley, CA, USA).

**Fig. 4:**
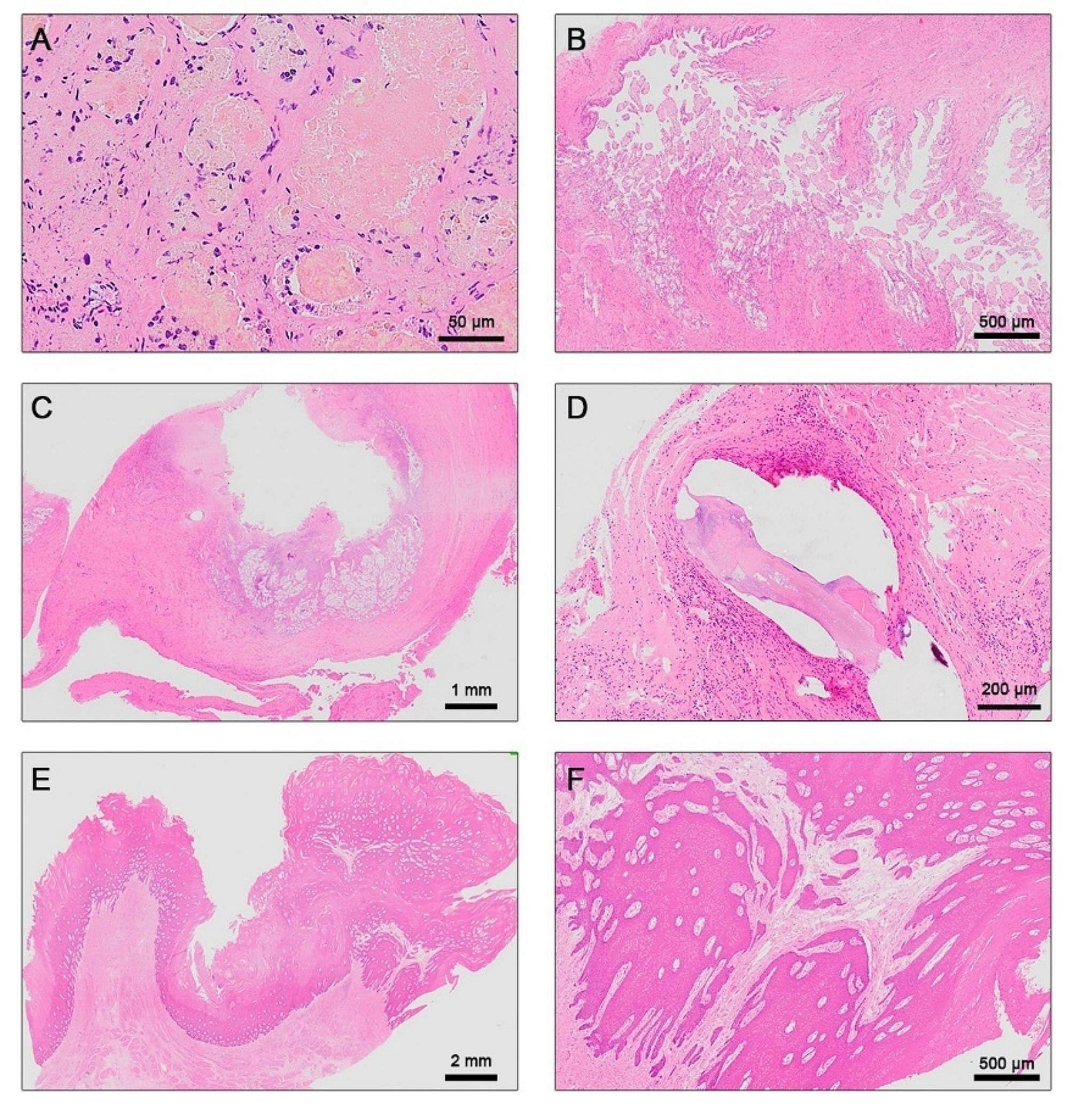
Histopathological examinations of the specimen. (**A**) A capsulated cyst near the right mammary gland was filled with dry proteinous material, presumed to be dried-up milk. (**B**) A uterine mass was assumed to be an endotheliochoral placenta. (**C**) A nodule in the liver shows highly fibrous surrounding tubular tissue with autolytic reaction. (**D**) Nodules in the lungs indicate a thick fibrous capsule with a more basophilic structure; elongated, round, or amorphous cysts were assumed to be encapsulated *Halocercus* sp. parasites. (**E**) Volcanic ulceration in the forestomach wall. Chronic ulcerative granulomatous gastritis was observed, with thick stratified squamous epithelium. (**F**) Enlargement of a volcanic ulceration. The stroma infiltrates into the squamous epithelium, resembling squamous papilloma

Several unusual nodules were observed in the mammary glands, uterus, liver, and lungs, prompting gross examination. Ulcerative gastritis lesions in the forestomach were subjected to histopathological analysis. In a capsulated cyst near the right mammary gland, the alveoli and excretory ducts were filled with dry proteinaceous material (Fig. 4A). In some alveoli, the epithelium appeared to be absent and macrophages that had phagocytosed the material were visible in the tissue between the alveoli. This cyst was presumed to be a post-lactation part of the mammary gland that still contained dried milk. The uterine mass was identified as a small, retained endotheliochoral placenta (Fig. 4B). A nodule in the liver exhibited highly fibrous surrounding tissue, forming a tubular structure (Fig. 4C). An autolytic reaction without active inflammation was observed at the periphery of the nodule. The granulomatous nodules in the lungs were characterized by necrotic debris and a thick fibrous capsule surrounding a central, more basophilic structure that could be elongated, round, or amorphous (Fig. 4D). These structural features strongly suggested the presence of encapsulated parasites (*Halocercus* sp.; Nematoda: Rhabditida: Pseudaliidae). Furthermore, chronic ulcerative granulomatous gastritis was observed in the forestomach wall, which was covered with thick stratified squamous epithelium forming papilloma-like structures covered with a parakeatosis surface (Fig. 4E). The stratified squamous epithelium in the central part of this lesion has been damaged by the parasite, leading to the loss of the epithelium layer itself, while the surrounding area has significantly thickened, forming ulcerations in a volcanic shape. Upon closer examination, it was evident that the stroma within the squamous epithelium resembled a squamous papilloma (Fig. 4F).

## Discussion and conclusions

This study provides evidence of the intake of fishing gear through radiological and pathological examinations in an Indo-Pacific finless porpoise (*N. phocaenoides*), which was stranded on Jeju Island, Republic of Korea.

The ingestion of foreign bodies has become increasingly common in cetaceans inhabiting Asian waters. The successful passage of foreign bodies through the gastrointestinal tract depends on various factors, such as their size, type, structure, and the number of swallowed bodies. Small foreign bodies that are ingested can often pass through the gastrointestinal tract without causing any symptoms or harm to the animals, eventually being excreted. However, in many cases, the ingestion of large, hook- or line-shaped foreign bodies can pose particular risks or even lead to death in cetaceans [23]. These objects are more likely to become lodged in the aerodigestive tract, resulting in a tract obstruction. Recently, PMCT has become more readily available for post-mortem imaging of cetaceans in Korean waters [23–25]. The built-in 3D volume-rendering function of PMCT can provide a 3D visualization of the morphology of high-density lodged items, such as fishing hooks and fish bones. This technique can offer an initial indication of the lodged object based on its morphology and the experience of the operator. Furthermore, spatial information of the obstructed site can be localized using the multiplanar reconstruction technique [23].

Although cetaceans have anatomically separate pathways for respiration and digestion, aspiration pneumonia, secondary to drowning, is still possible as a result of repeated regurgitation due to ingestion of a foreign body [23, 26–28]. Radiological signs presented in the Indo-Pacific finless porpoise on which this case report is based are consistent with wet drowning secondary to pulmonary effusion. Pulmonary effusion, resulting from seawater aspiration, is characterized by widespread GGO, bronchial wall thickening, blunting of the costophrenic angle, and bronchiectasis. GGO in the lungs with a mosaic pattern is considered a typical, though not highly specific, finding of drowning. Admittedly, pulmonary effusion due to respiratory distress (agony) during suffocation could not be ruled out. Zhu and his team [29] suggest that the amount of intra-alveolar granular staining may be a possible indicator of agony. Nonetheless, intra-alveolar granular staining was not observed in our case despite possible freezing artifacts. With the evidence of bronchial wall thickening and blunting of the costophrenic angle, we could summarise that this carcass is likely to experience drowning before death. GGO in the lung parenchyma can lead to increased pulmonary opacity due to reduced air content, without obliterating the underlying bronchial, alveolar, and vascular margins [30]. The mosaic attenuation patterns in the lungs result from the uneven distribution of hypo-perfused and hyper-perfused areas. This distribution may occur due to fluid aspiration followed by bronchospasm, which scatters more multifocally throughout the lung. PMCT provides a non-invasive and objective documentation of the pulmonary condition in cetaceans, which offers useful information to complement the findings of conventional necropsy.

The porpoise reported in the present study has been influenced by two types of human activities. Firstly, the ingestion of angling gear; these marine litter items were assumed to be angling gear designed for catching Jeju hairtail (*T. lepturus*) [31]. It is hypothesized that the porpoise attempted to sever the fishing lines and ingest hairtails attached to the hooks or the porpoise caught the fish that were incidentally hooked and may have eventually broken the nylon line through force, as evidenced by the discovery of intact gear components in the forestomach. The bait loss rate of hairtail gear can be from 20 to 100% depending on the time of immersion, the number of operations, and the hook numbers [32], and thus, the high loss rate of artificial baits and hooks and depredation present an environmental and industrial fishing problem in Jeju seawater. The hooks in this specimen exhibited no rust discoloration, suggesting they were ingested shortly before the individual’s death. The forestomach is a chamber where food is stored without the secretion of digestive fluids. Even though the rate at which iron oxide can be produced is unknown, it will likely take a longer time for rust to form in the forestomach due to oxidation processes between dissolved oxygen in the water and the iron in the hooks [33, 34]. These fishing hooks were tangled in a round shape along the fishing lines and surrounded by numerous adult *Anisakis* worms. Hairtails are typical intermediate hosts of the nematode [35, 36], and the cetaceans are also a typical definitive host of them [37]. The sequence of events between the ingestion of fishing hooks and the infection by *Anisakis* sp., as well as any potential correlation between them, remains unverified and warrants further investigation.

Nonetheless, regardless of the sequence of events, it was hypothesized that the symptoms would significantly influence each other. Due to the presence of foreign bodies in the forestomach, porpoises may suffer from eating disorders, chronic pain, and malnutrition, which can lead to a compromised immune system [38]. The larvae or cysts of *Anisakis* sp. in the food contents might have been in an ideal condition to grow because of the longer retention time of the food, the inflammatory response in the stomach, and the existence of foreign bodies that provide a sheltered environment. As shown in previous studies [39–41], nematode superinfection leads to ulcerative gastritis and aggravated digestive deconditioning.

On the other hand, given the severity of *Anisakis* sp. found in the oral cavity, esophagus, and forestomach, it is plausible that this porpoise had been infected with parasites from infected prey sources long, suggesting the possibility of these parasites breeding within the stomach over an extended period before ingesting fishing gear. Severe gastrointestinal parasitic infections could lead to indigestion and lethargy [42, 43], potentially making the porpoise more likely to depredate the caught prey by fishing, ingest the fishing gear, and be caught by net ships that easily obtain the organisms being targeted.

The second human activity that affected the porpoise may have been net fishing. Suffocation could be a cause of death in this specimen. The most common cause of suffocation in cetaceans is being bycaught in nets [44], which may also have been the case in this individual. Many studies have shown that bycatching has a significant effect on cetacean populations in Korea [45–47]. This specimen, in particular, may serve as a crucial example to emphasize the severity of ingestion of artificial foreign bodies with depredation and bycatching in Korea, because its death was influenced by the impact of human fishery. Based on the histopathological results of the mammary gland and the retained placenta in the uterus, the porpoise was assumed to be in the early stages after delivering her calf. This case underscores the importance of addressing the severity of the impact of fishing activities on marine mammals in Korean waters.

In March, all the seawater around Jeju Island forms legal fishing grounds for hairtail, with a particularly high concentration of fishing activity along the eastern sea of Jeju [48]. It is possible that hairtail fishing gear ingestion occurred in these eastern waters. Considering that the fresh specimen was identified with Smithsonian code 2, it can be postulated that the porpoise likely experienced being bycaught near Jeju Island. *Neophocaena phocaenoides* inhabits a vast region ranging from the Persian Gulf in the west to the coasts of India and the South China Sea near Taiwan in the east [49], but previous research did not include Korean waters as part of the species distribution, despite rare reports of its detection here. The number of cases of this species beached and discovered on the shores of Jeju Island has gradually increased in recent years. Rising sea temperatures and the tropicalization of oceans, which are attributed to global warming, are anticipated to bring significant changes to marine environments worldwide [50, 51], including the seas surrounding Jeju Island, the southern coast, and Ulleung Island in South Korea [52–54]. These changes are likely to have profound effects on the habitats of *N. phocaenoides* throughout its range. Further studies are also needed to determine whether *N. phocaenoides* is resident in Jeju seawater and the intermediate areas between Jeju and its easternmost range in Taiwan, specifically in the waters around Japan and eastern China.

## Data Availability

All data and materials are within this published paper.

## Abbreviations

ALDFG: Abandoned, lost, or discarded fishing gear
PMCT: Post-mortem computed tomography
CT: Computed tomography
sFOV: The scanned field of view
DICOM: Digital Imaging and Communications in Medicine
FIRA: Fisheries Resources Agency
BCS: Body condition score
H&E: Hematoxylin and eosin

## References

[1] Phillips RA, Ridley C, Reid K, Pugh PJ, Tuck GN, Harrison N (2010). Ingestion of fishing gear and entanglements of seabirds: monitoring and implications for management. Biol Conserv.

[2] Mitchell JD, McLean DL, Collin SP, Langlois TJ (2019). Shark depredation and behavioural interactions with fishing gear in a recreational fishery in Western Australia. Mar Ecol Prog Ser.

[3] Lauriano G, Caramanna L, Scarno M, Andaloro F (2009). An overview of dolphin depredation in Italian artisanal fisheries. J Mar Biol Assoc U K.

[4] Casselberry GA, Markowitz EM, Alves K, Russo JD, Skomal GB, Danylchuk AJ (2022). When fishing bites: understanding angler responses to shark depredation. Fish Res.

[5] Hamilton S, Baker GB (2019). Technical mitigation to reduce marine mammal bycatch and entanglement in commercial fishing gear: lessons learnt and future directions. Rev Fish Biol Fish.

[6] El-Khaled YC, Duarte CM, Peixoto RS (2023). Evidence of hawksbill turtle (*Eretmochelys imbricata*) depredation on fish caught in gillnets. Front Mar Sci.

[7] Kammann U, Nogueira P, Wilhelm E, Int-Veen I, Aust MO, Wysujack K (2023). Abandoned, lost or otherwise discarded fishing gear (ALDFG) as part of marine litter at the seafloor of the Baltic Sea–Characterization, quantification, polymer composition and possible impact. Mar Pollut Bull.

[8] Richardson K, Asmutis-Silvia R, Drinkwin J, Gilardi KV, Giskes I, Jones G, O’Brien K, Pragnell-Raasch H, Ludwig L, Antonelis K (2019). Building evidence around ghost gear: global trends and analysis for sustainable solutions at scale. Mar Pollut Bull.

[9] Kim S, Lee W, Moon Y (2014). The estimation of derelict fishing gear in the coastal waters of South Korea: trap and gill-net fisheries. Mar Policy.

[10] Jo K, Im J, Park BY, Cho B, Joo S, Kim BY, Kim T (2022). Possible link between derelict fishing gear and sea turtle strandings in coastal areas. Mar Pollut Bull.

[11] Stelfox M, Hudgins J, Sweet M (2016). A review of ghost gear entanglement amongst marine mammals, reptiles and elasmobranchs. Mar Pollut Bull.

[12] Votier SC, Archibald K, Morgan G, Morgan L (2011). The use of plastic debris as nesting material by a colonial seabird and associated entanglement mortality. Mar Poll Bull.

[13] Padula AD, Machado R, Milmann L, de León MC, Gana JC, Wickert JC, Argañaraz ME, Bastida RO, Rodríguez DH, Denuncio PE (2023). Marine debris ingestion by odontocete species from the Southwest Atlantic Ocean: absence also matter. Mar Poll Bull.

[14] Brownell RL, Reeves RR, Read AJ, Smith BD, Thomas PO, Ralls K (2019). Bycatch in gillnet fisheries threatens critically endangered small cetaceans and other aquatic megafauna. Endanger Species Res.

[15] Unger B, Rebolledo EL, Deaville R, Gröne A, IJsseldijk LL, Leopold MF, Siebert U, Spitz J, Wohlsein P, Herr H (2016). Large amounts of marine debris found in sperm whales stranded along the North Sea coast in early 2016. Mar Pollut Bull.

[16] Heezen BC (1957). Whales entangled in deep sea cables. Deep Sea Res.

[17] Timóteo S, Albrecht J, Rumeu B, Norte AC, Traveset A, Frost CM (2023). Tripartite networks show that keystone species can multitask. Funct Ecol.

[18] Jefferson TA, Wang JY (2011). Revision of the taxonomy of finless porpoises (genus *Neophocaena*): the existence of two species. J Mar Anim Ecol.

[19] Ku JE, Choi SG (2022). Population structure of Finless Porpoise (*Neophocaena phocaenoides*) discovered off Coastal Waters, Republic of Korea. Genes.

[20] per Geraci JR, Lounsbury VJ, Yates N. Marine mammals ashore: a field guide for strandings. Second Edition. National Aquarium in Baltimore. 2005. https://api.semanticscholar.org/CorpusID:127512602. Accessed 24 Mar 2005.

[21] Joblon MJ, Pokras MA, Morse B, Harry CT, Rose KS, Sharp SM (2014). Body condition scoring system for delphinids based on short-beaked common dolphins (*Delphinus delphis*). J Mar Anim Ecol.

[22] Hrabar J, Bočina I, Kurilj AG, Đuras M, Mladineo I (2017). Gastric lesions in dolphins stranded along the Eastern Adriatic coast. Dis Aquat Organ.

[23] Yuen AHL, Lee SB, Kim SW, Lee YM, Kim DG, Poon CTC et al. Fatal upper aerodigestive tract obstruction in an east Asian finless porpoise (*Neophocaena asiaeorientalis sunameri*): findings in post-mortem computed tomography. Forensic Sci Med Pathol, 2023;1–8. 10.1007/s12024-023-00732-0 37831312

[24] Yuen AHL, Kim SW, Lee SB, Lee S, Lee YR, Kim SM (2022). Radiological Investigation of Gas Embolism in the east Asian finless porpoise (Neophocaena asiaeorientalis sunameri). Front Mar Sci.

[25] Lee SB, Yuen AHL, Lee YM, Kim SW, Kim S, Poon CTC (2023). Adhesive bowel obstruction (ABO) in a stranded narrow-Ridged Finless Porpoise (Neophocaena asiaeorientalis sunameri). Animals.

[26] Stoskopf MK. Marine Mammals. Environmental Diseases. Ed; Aiello SE, In: The Merck Veterinary Manual. 8th ed. Philadelphia, National Publishing Inc; 1998; pp. 1359.

[27] Costidis AM, Berman M, Cole T, Knowlton A, McLellan WA, Neilson J (2013). Sharp trauma induced by vessel collision with pinnipeds and cetaceans. Dis Aquat Organ.

[28] IJsseldijk LL, Scheidat M, Siemensma ML, Couperus B, Leopold MF, Morel M (2021). Challenges in the assessment of bycatch: postmortem findings in harbor porpoises (Phocoena phocoena) retrieved from gillnets. Vet Pathol.

[29] Zhu BL, Ishida K, Quan L, Fujita MQ, Maeda H (2000). Immunohistochemistry of pulmonary surfactant apoprotein A in forensic autopsy: reassessment in relation to the causes of death. Forensic Sci Int.

[30] Banday SA, Nahvi R, Mir AH, Khan S, AlGhamdi AS (2022). Alshamrani. Ground glass opacity detection and segmentation using CT images: an image statistics framework. IET Image Proc.

[31] An YI, He P, Arimoto T, Jang UJ (2017). Catch performance and fuel consumption of LED fishing lamps in the Korea hairtail angling fishery. Fish Sci.

[32] Kim BY, Park YS, Lee CH (2009). Hooking rate and bait loss rate of traditional hairtail hand line according to immersion time in the coastal waters of Jeju. J Korean Soc Fish Ocean Technol.

[33] Klemens L, Neven CJ, Bär T, Krumme U, Dähne M (2022). In vitro forestomach digestion experiments give less-biased estimates of food composition in odontocetes. Biol Open.

[34] Klepper C, Oliveira SB (2020). Hematite ingestion in pediatrics: risk of iron poisoning. J Pediatr Gastroenterol Nutr.

[35] Kim JH, Nam WH, Jeon CH (2016). Genetic identification of anisakid nematodes isolated from largehead hairtail (*Trichiurus japonicus*) in Korea. Fish Aquat Sci.

[36] Umehara A, Kawakami Y, Ooi HK, Uchida A, Ohmae H, Sugiyama H (2010). Molecular identification of *Anisakis* type I larvae isolated from hairtail fish off the coasts of Taiwan and Japan. Int J Food Microbiol.

[37] Shiozaki A, Amano M (2017). Population-and growth-related differences in helminthic fauna of finless porpoises (*Neophocaena asiaeorientalis*) in five Japanese populations. J Vet Med Sci.

[38] Lisičić-Konaković M, Kulašević A, Melunović M. Ingestion of a metallic foreign body (hair clip) by a small child. Paediatr Croat. 2022;66.

[39] Pons-Bordas C, Hazenberg A, Hernandez-Gonzalez A, Pool RV, Covelo P, Sánchez-Hermosin P, … Aznar FJ. Recent increase of ulcerative lesions caused by *Anisakis* spp. in cetaceans from the north-east Atlantic. J Helminthol. 2020;94:e127. 10.1017/S0022149X20000115 32100663

[40] Katahira H, Matsuda A, Banzai A, Eguchi Y, Matsuishi TF (2021). Gastric ulceration caused by genetically identified *Anisakis simplex* sensu stricto in a harbor porpoise from the Western Pacific stock. Parasitol Int.

[41] Gomes TL, Quiazon KM, Kotake M, Fujise Y, Ohizumi H, Itoh N, Yoshinaga T (2021). *Anisakis* spp. in toothed and baleen whales from Japanese waters with notes on their potential role as biological tags. Parasitol Int.

[42] Saravanan S, Dinakaran AM, Muralidharan J, Geetha M, Selvaraju G (2009). Prevalence of subclinical gastrointestinal parasitic infection in dairy animals. Indian J Field Vet.

[43] Jasti A, Ojha SC, Singh YI (2007). Mental and behavioral effects of parasitic infections: a review. Nepal Med Coll J.

[44] Dolman SJ, Moore MJ. Welfare implications of cetacean bycatch and entanglements. Mar Mammal Welfare: Hum Induc Change Mar Environ Its Impacts Mar Mammal Welf 2017;41–65.

[45] Kim DN, Sohn H, An YR, Park KJ, Kim HW, Ahn SE, An DH (2013). Status of the cetacean bycatch near Korean waters. Korean J Fish Aquat Sci.

[46] Lee S, Choi S, Kim JH, Kim HW, Sohn H (2018). Characteristics of the cetacean bycatch in Korean coastal waters from 2011 to 2017. Korean J Fish Aquat Sci.

[47] Song KJ (2018). Bycatch of cetaceans in Korea fisheries in the East Sea. Fish Res.

[48] Owiredu SA, Kim KI (2021). Spatio-temporal fish catch assessments using fishing vessel trajectories and coastal fish landing data from around Jeju Island. Sustainability.

[49] Jefferson TA, Wang JY (2011). Revision of the taxonomy of finless porpoises (genus *Neophocaena*): the existence of two species. J Mar Anim Their Ecol.

[50] Freitas D, Arenas F, Vale CG, Pinto IS, Borges D (2023). Warning of warming limpets: sea temperature effects upon intertidal rocky assemblages. J Mar Biolog Assoc UK.

[51] Yang M, Qiu Y, Huang L, Cheng M, Chen J, Cheng B, Jiang Z (2023). Changes in sea surface temperature and sea ice concentration in the Arctic Ocean over the past two decades. Remote Sens.

[52] Lee KT, Perrois G, Yang HS, Kim T, Choi SK, Kang DH, Kim T (2023). Impact of super typhoon ‘Hinnamnor’ on density of kelp forest and associated benthic communities in Jeju Island, Republic of Korea. J Mar Sci Eng.

[53] Kang Y, Lee DH. Coastal warming heightens direct impacts of seawater temperature on nutrients near aquaculture farms in Korea. Sci Total Environ. 2023:164643. 10.1016/j.scitotenv.2023.164643 37271382

[54] Park KW, Chung MH, Yoo MH, O KS, Kim KY, Park TG, Young SH (2023). Impact of phytoplankton community structure changes in the South Sea of Korea on marine ecosystems due to climate change. Water.

